# Patient education in chronic heart failure in primary care (ETIC) and its impact on patient quality of life: design of a cluster randomised trial

**DOI:** 10.1186/s12875-014-0208-3

**Published:** 2014-12-24

**Authors:** Hélène Vaillant-Roussel, Catherine Laporte, Bruno Pereira, Gilles Tanguy, Jean Cassagnes, Marc Ruivard, Gilles Clément, Jean-Yves Le Reste, Jean-Pierre Lebeau, Jean-François Chenot, Denis Pouchain, Claude Dubray, Philippe Vorilhon

**Affiliations:** General Practice Department, Faculty of Medicine of Clermont-Ferrand University, 28 Place Henri Dunant, 63000 Clermont-Ferrand, France; Clinical Investigation Center, INSERM CIC 501, Clermont-Ferrand University Hospital, 58 Rue Montalembert, 63000 Clermont-Ferrand, France; EA 7280 NPsy-Sydo, University of Auvergne, Faculty of Medicine of Clermont-Ferrand, 28 Place Henri Dunant, 63000 Clermont-Ferrand, France; Biostatistics unit, Clinical Research and Innovation Department, Clermont-Ferrand University Hospital, 58 Rue Montalembert, 63000 Clermont-Ferrand, France; Cardiology Department, Clermont-Ferrand University Hospital, 58 Rue Montalembert, 63000 Clermont-Ferrand, France; Internal Medicine Department, Clermont-Ferrand University Hospital, Place Lucie et Raymond Aubrac, 63000 Clermont-Ferrand, France; General Practice Department, Faculty of Medicine of Brest University, 22 avenue Camille Desmoulins, 29238 Brest, France; General Practice Department, Faculty of Medicine of Tours University, 10 boulevard Tonnellé, 37032 Tours, France; General Practice Department, Institute of Community Medicine, University of Greifswald, Fleischmannstr. 42-44, 17475 Greifswald, Germany; EA 4681PEPRADE, University of Auvergne, Faculty of Medicine of Clermont-Ferrand, 28 Place Henri Dunant, 63000 Clermont-Ferrand, France

**Keywords:** Heart failure, Quality of life, Patient education, Primary care, Cluster-randomised controlled trial

## Abstract

**Background:**

Chronic heart failure, is increasing due to the aging population and improvements in heart disease detection and management. The prevalence is estimated at ~10% of the French general practice patient population over 59 years old. The primary objective of this study is to improve the quality of life for heart failure patients though a complex intervention involving patient and general practitioner (GP) education in primary care.

**Methods:**

A randomised, cluster controlled trial, stratified over 4 areas of the Auvergne region in France comparing intervention and control groups. The inclusion criteria are: patients older than 50 years with New York Heart Association (NYHA) stage I, II, or III heart failure, with reduced ejection fraction or with preserved ejection fraction. Heart failure should be confirmed by the patient’s cardiologist according to the European Society of Cardiology guidelines criteria. The exclusion criteria include: severe cognitive disorders, living in an institution, participating in another clinical trial, having NYHA stage IV heart failure, or a lack of French language skills. The complex intervention consists of training at the GP practice with an interactive 2-day workshop to provide a patient’s education programme. GPs are trained to perform case management, lifestyle counselling and motivational interviewing, to educate patients on the main topics including clinical alarm signs, physical activity, diet and cardiovascular risk factors. The patients’ education sessions are scheduled at 1, 4, 7, 10, 13 and 19 months following the start of the trial. The primary outcome to be assessed is the impact on the quality of life as determined using two questionnaires: the Minnesota Living with Heart Failure Questionnaire and SF-36. To detect a difference in the mean quality of life at 19 months, we anticipate studying a minimum of 400 patients from 80 GPs.

**Discussion:**

This trial will provide insight into the effectiveness of a complex intervention to educate patients with heart failure including a 2-day GP workshop and patients’ education programme in the setting of a GP consultation to improve the quality of life in patients with chronic heart failure. This complex intervention tool could be used during initial and further medical training.

**Trial registration:**

ETIC is a cluster-randomised, controlled trial registered on ClinicalTrials.gov [NCT01065142, 2010, Feb 8] and the French drug agency [Agence Nationale de Sécurité du Médicament et des produits de santé; registration number: 2009-A01142-55, on March 5th, 2010].

**Electronic supplementary material:**

The online version of this article (doi:10.1186/s12875-014-0208-3) contains supplementary material, which is available to authorized users.

## Background

Chronic heart failure (CHF) is a common pathology increasing in prevalence due to the aging of the population and detection and improvements in heart disease management [1]. Approximately 10% of the French general practice patient population over 59 years old [2] has CHF. Patients with CHF have poor quality of life due to their symptoms and recurrent hospitalisations, and due to its high complication rates CHF is expensive to treat [3]. Every year in France there are approximately 200 000 hospitalisations due to CHF which accounts for 1.5% of global health expenditure [4]. The European Society of Cardiology guidelines recommend pharmacological therapy to reduce morbidity, mortality and improve patient quality of life; and non-pharmacological management (self-care, behavioural and patient education) to improve adherence to treatment and quality of life [5].

The results from a meta-analysis have shown that the implementation of comprehensive disease management programmes leads to a significant reduction in mortality (odds-ratio (OR) = 0.80), reduced hospitalisation for cardiac failure (OR = 0.76) and all illnesses (OR = 0.58) [6]. In France, some studies have assessed the impact of education for patients with CHF [7,8]. The interventions were delivered by multidisciplinary teams, including outpatient clinics attached to hospitals, which does not reflect the French primary care setting where most general practitioners (GPs) work single-handed. There have been very few studies exclusively focused on assessing the effect of CHF management programmes in a primary care setting, but several including primary care situations amongst others [9-13]. Other studies have been conducted in primary care but the interventions were not carried out by the GPs themselves: i.e. patient recruitment occurred in primary care but the intervention was conducted by nurses or doctors’ assistants [14,15]. More evidence is needed in primary care because the generalisability of hospital- or outpatient-based programmes to primary care is limited.

Therefore we want to assess the effectiveness of a complex intervention that incorporates education of patients with CHF by GP’s trained to promote self-care management and behaviour management. The aim of the ETIC trial (Education Thérapeutique des patients Insuffisants Cardiaques/Therapeutic Education for patients with Cardiac Failure) is to improve the quality of life of patients with heart failure though a complex intervention involving patient and GP education in primary care. The secondary objectives are to assess the effects of the training on: all-cause and heart-failure (HF)-associated mortality; all-cause and HF-associated hospitalisation; the cumulative number of all cause death and HF hospitalisations; adherence to treatment; changes in NYHA heart failure stage; changes weight and body mass index (BMI) and treatment for a follow-up period of 19 months.

## Methods

### Study design and randomisation

**Figure 1:**
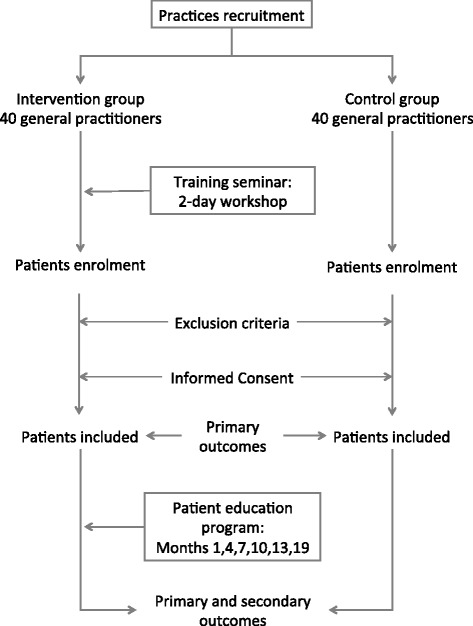
**Overview of ETIC study protocol.**

**Figure 2:**
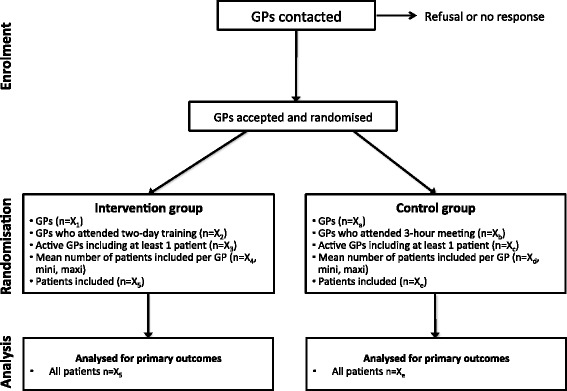
**ETIC trial flow chart.**

The global study design is presented in Figure 1. ETIC is a cluster-randomised, controlled clinical trial with general practices as the unit of randomisation (Figure 2). The randomisation list was drawn up with the software Stata, version 10 (StataCorp, College Station, Texas, US) by the biostatistician before the start of the trial. A cluster design was chosen for pragmatic reasons and to avoid contamination bias. All GPs who volunteered were randomised and the patients did not know to which group their GP had been assigned. GPs that were located within the same practice represented a cluster and such GPs were placed in the same group to avoid cross-contamination. The trial is being carried out across the Auvergne in France, with stratification according to the 4 departments (administrative areas) in this region.

### Inclusion and exclusion criteria

#### Inclusion criteria

Patients older >50 years, with New York Heart Association (NYHA) stage I, II or III CHF with reduced ejection fraction (HFrEF) or preserved ejection fraction (HFpEF) are eligible for inclusion. Heart failure has to be documented and confirmed by the patient’s cardiologist according to the European Society of Cardiology guidelines, namely, patients have, currently or in the past, symptoms, signs of fluid retention and objective evidence of an abnormality of the structure or function of the heart at rest confirmed by echocardiographic criteria [5]. When GPs recruit patients, patients’ cardiologists are sent a letter to ask them to confirm heart failure according these guidelines.

#### Exclusion criteria

Patients that suffer from severe cognitive disorders (as judged by the GP), who are living in an institution at the time of inclusion, are participating in another clinical trial, have NYHA stage IV heart failure, or a lack of French language skills are excluded from participating in the trial.

### Instruments and outcomes

The primary outcome is the patients’ quality of life as measured by SF-36 [16] and the Minnesota Living with Heart Failure Questionnaire (MLHFQ) [17]. The SF-36 is a generic measure of health impairment, with scores ranging from 0 to 100: zero indicates the worst quality of life and 100 the best. The MLHFQ is specific for chronic heart failure and has 21 questions with scores ranging from 0 to 105: zero indicates best quality of life and 105 the worst. Quality of life is assessed at baseline and at 7, 13 and 19 months by the patients, or their main caregiver, within 7 days of their appointment with their GP. A score of < 24 on the MLHFQ signifies a good quality of life, a score between 24 and 45 signifies a moderate quality of life, and a score > 45 signifies a poor quality of life [18]. We will assess these cut-off scores to interpret results in clinical terms. We were unable to find published cut-off information for the SF-36 health scores.

The secondary outcomes are: all-cause and heart-failure-associated mortality; all-cause and heart-failure-associated hospitalization, number of days in hospital; the cumulative number of all cause death or HF hospitalizations and the cumulative number of days of hospitalisation; adherence with therapy; evolution of NYHA heart failure stage; evolution of treatment; and changes in weight and body mass index (BMI) at 19 months. We will assess guideline adherence of treatment at baseline and during the follow-up period. Patient adherence with therapy will be assessed by the patients themselves with a self-administrated questionnaire (validated in French) at baseline and at the end of follow-up period [19].

Should the planned follow-up appointment be missed, the reason is identified and categorised as follows: lost-to-follow-up; death; unable to attend for another reason. Hospitalisations and mortality will be recorded by GPs at each planned visiting case report form.

### GP recruitment and patient enrolment

GPs are eligible to participate in the trial if they provide in their practice standard care as opposed to exclusively providing alternative medicine such as acupuncture or homeopathy. Participating GPs must sign an informed consent form and agree to implement the study protocol. Potentially eligible GPs are identified via the Regional Primary Care Physician Association with an address database.

In both groups, GPs identify eligible CHF patients and ask them to participate. The GPs recruit the first eligible patients to participate in the clinical trial until they have included at least five patients. The inclusion period lasts one year. The GPs in the two groups receive the same compensation for their participation. The GP shares an information sheet and a consent form with the patients. During the inclusion visit, the GP explains the aim of the clinical trial, i.e. understand and improve treatment of patients with heart failure, without describing the objectives. Patients willing to participate sign the consent form at home within seven days of the visit and send it to the data management centre with the first two quality of life questionnaires.

### Intervention group

No medication is tested in this trial and the GPs are allowed to adapt patients’ treatment if necessary. The complex intervention consists of training at the GP practice with an interactive 2-day workshop to provide a patient’s education programme.

#### GP training

At an interactive 2-day workshop, GPs assigned to the intervention group are trained to perform case management and educate patients (Table 1). The two-day workshop includes the following elements: information on the case report forms and the patients’ inclusion/exclusion criteria, information on CHF (epidemiology, NYHA stages, treatment, patient adherence etc.) based on The European Society of Cardiology guidelines [5], information on patient education concepts, principles of GP-patient-communication, lifestyle counselling and motivational interviewing based on the 5As model (ask, assess, advise, assist and arrange) [20] and the use of several case vignettes simulating GP- patient consultations. GPs are trained to educate patients on the main topics of clinical alarm signs, physical activity, diet and cardiovascular risk factors. During the 2-day workshop, GPs are trained by experts: a nutritionist, an endocrinologist, a cardiologist, three GPs and a pharmacist, who are all patient education trainers and one has a Masters degree in patient education.

**Table 1:** **Training seminar for general practitioners: 2-day workshop**

Module 1: Introduction	- Introduction to the concepts of the ETIC trial and patient education
Module 2: Heart failure	- Chronic heart failure: definitions; epidemiology; clinical diagnosis; treatment guidelines∗; echocardiographic criteria; cardiac biomarkers: B-type natriuretic peptide (BNP) and NT-proBNP (how, when to prescribe them, how to use them)
	- Clinical symptoms: how to recognize heart failure in daily practice?
	- NYHA stages: definitions, assessment of NYHA stages from case vignettes
	- Suspicious clinical signs
	- Adaptation of physical activity as a function of the NYHA stage
Module 3: Concepts of patient education	- Assessment and building on the patient’s previous knowledge
	- Identification of life-style and dietary habits, physical activity, hobbies, leisure activities, projects, and resources available to the patient
	- Assessment of the patient’s stage of change, motivation and attitudes
	- Collaboration with the patient to define achievable and measurable objectives
Module 4: Communication	- Communication skills
	- Communication tools
	- Motivational interviewing
	- Lifestyle counselling based on the 5As model (ask, assess, advise, assist, and arrange)
Module 5: Role-playing to simulate a patient consultation with the general practitioner	- Identification and use of the patient’s knowledge (clinical alarm signs, physical activity, diet and cardiovascular risk factors), values, motivation, projects and resources to implicate the patient in their personal objectives
	- Classification of the personal objectives by therapeutic priority and patient preference
	- Use of effective communication strategies
Module 6: Case report forms	- Inclusion and exclusion criteria
	- How to promote and present the ETIC trial to patients
	- How to fill in the case report forms
	- How to organize the follow-up and topics: educational booklet and educational tools (i.e. dietary leaflets, clinical alarm signs)

∗Dickstein K, Cohen-Solal A, Filippatos G, McMurray JJ, Ponikowski P, Poole-Wilson PA, Strömberg A, van Veldhuisen DJ, Atar D, Hoes AW, Keren A, Mebazaa A, Nieminen M, Priori SG, Swedberg K; ESC Committee for Practice Guidelines (CPG). **ESC Guidelines for the diagnosis and treatment of acute and chronic heart failure 2008: the Task Force for the Diagnosis and Treatment of Acute and Chronic Heart Failure 2008 of the European Society of Cardiology. Developed in collaboration with the Heart Failure Association of the ESC (HFA) and endorsed by the European Society of Intensive Care Medicine (ESICM).** Eur Heart J. 2008;**29**(19):2388-442.

#### Patient’s intervention level

The patients’ education sessions will be performed by their own GPs. The education sessions are standardised both in their timing, every three months (Figure 1), and topics covered (Table 2). GPs have an education booklet in their case report form with the topics covered and education tools (ie dietary leaflets, clinical alarm signs) (Additional file 1). The first educational session (educational diagnosis) for the patient occurs at month one (Figure 1) and covers several topics: life-style and dietary habits, physical activity, hobbies, leisure activities, projects and details resources available for patients (Additional file 2). This first step is necessary to establish existing knowledge, attitudes and motivation. Patients have four further education sessions at 4, 7, 10 and 13 months followed by an overview session six months after the last education session at month 19.

**Table 2:** **Education intervention topics**

**Knowledge, attitudes and motivation**	- Do you suffer from heart failure?
	- What is « heart failure » for you?
	- What do you know about heart failure?
	- How do you live with this disease?
	- What impact heart failure has on your life (personal, professional, social)?
	- What are your fears?
	-What are your expectations?
**Clinical alarm signs**	- For you, what could be a clinical alarm sign of your heart failure?
	- What should you do to detect clinical alarm signs?
	- Do you know what to do if you detect clinical alarm signs?
**Physical activity**	- What does physical activity mean for you?
	- What is yours? Household?, leisure (e.g. gardening)? Transportation (e.g. walking, car)?
	- When are you breathless? (NYHA)?
	- What would you be ready to change in your habits?
**Diet**	- Where do you eat yours meals?
	- Who does the cooking?
	- High salt food: what do you know about? What is your comsumption? What is your point of view, what changes are you ready to start?
	- BMI ≥ 30 : diet mistakes (snack food, overeating) or diet troubles ?
	- BMI ≤ 18 adult patients or 21 ealderly patients : diet mistakes or diet troubles ?

There is no predetermined order, each theme is evoked depending on the patients’ needs and based on the first education session (Additional file 2).

Patient education sessions (at 4, 7, 10, 13, 19 months) are based on patient’s experience and knowledge of their illness, including clinical alarm signs, physical activity, diet and cardiovascular risk factors. Patient education sessions are adapted for each patient, based on the first education session, one month after inclusion and at each of the following visits to match the needs and motivation of each patient. As this is a pragmatic trial aiming to assess the impact of an educational programme in daily clinical practice, the delivery of the program is allowed to vary between health care providers. The health care providers have the flexibility to adapt the programme according to patients’ needs. For example, if a patient smokes but does not want to discuss this, this is respected but, at the following visit, the patient is asked again if they want to discuss their smoking habits [21]. After each education session, GPs write in their case report form which topics they explored (Additional file 3). At the end of each visit, patients fix their own personal objectives with the GP, for example, only eat cooked pork meat products twice a week, verify weight once a week and walk their dog once a week (Additional file 3). These objectives are evaluated at the following patient education session and can be modified and adapted at each visit. This continuous evaluation of patients’ needs and objectives provides the basis for further education sessions. The GPs are trained to manage their own education objectives (i.e. HTA, diet, adherence, etc.) and patient objectives (i.e. to be able to walk their grandchildren to school). They simulate several patient education sessions during the 2-day workshop. GPs have case report forms with written standardised instructions for the programme patient education sessions and to summarise each consultation and write personal patient objectives.

### Control group

The GPs in the control group attend a three-hour information evening session to learn about the case report forms and the patients’ inclusion/exclusion criteria. Their patients have the same schedule for visits as those in the intervention group (at 1, 4, 7, 10, 13, 19 months) but without specific education intervention.

### Statistical considerations

#### Sample size calculation and power analysis

The sample size required must accommodate the need to detect a difference between the intervention and control groups in the mean quality of life (sample size estimated for each domain of SF-36 and for the MLHFQ score) of 12 points with 90% power and 5% two-sided type 1 error taking into account clustering by practice (intra-cluster correlation considered to be between 0.10 and 0.20) [22], and an estimated 20% dropout rate for GPs and patients. As there is negligible information in the literature regarding SF-36 and MLHFQ assessment in intervention studies, a difference of 12 points was chosen using Brotons’ work [23] and the recommendations of Cohen [24] regarding variability of the quality of life indicators [16]. Estimation of the effect size (ES) can be achieved by a literature search, expert knowledge or using pilot studies. It is also possible to explore several scenarios using conventional effect sizes: small (ES = 0.2), medium (ES = 0.5) and high (ES = 0.8). Variability in the quality of life indicators is fixed for MLHFQ and SF-36 and corresponds to an effect size of ≥ 0.6 [17,24]. With these simulations, it is estimated that 40 general practices recruiting five patients each are required per group, therefore 200 patients per group. Overall, 400 patients from 80 GPs will be recruited.

#### Statistical analysis

The statistician will be blinded with regard to treatment allocation. The responses to the questionnaire will be entered into a customised Access database (Microsoft, Redmond, Wash, US). Analyses will be made using Stata, version 13 (StataCorp, College Station, Tex, US). If necessary, an adjustment to accommodate differences between the baseline characteristics of the groups will be made. Continuous data will be presented as mean ± standard deviation (SD). The comparisons between study groups will be analysed using Chi-square or Fisher's exact test for categorical variables, and Student's or Mann–Whitney tests for quantitative variables, with normality verified by the Shapiro-Wilk test and homoscedasticity by the Fisher-Snedecor test. The correlation between SF-36 and MLHFQ will be assessed using Pearson’s correlation coefficient. Hierarchical linear regression models with random-effects levels for practice, individuals within practices and repeated measurements per individual (slope and intercept) will be used to estimate the effects of the intervention on SF-36 and MLHFQ scores for the post-baseline time points. These models will include an interaction factor between both the randomisation group and the particular time point, and will be adjusted for baseline SF-36 scores, age, gender, diagnosis, smoking history, treatment and socioeconomic status. Intra-class correlation coefficients will be presented by group. The secondary analyses will compare deaths (overall and due to heart failure) using Kaplan-Meier estimation and the Cox proportional-hazards regression model, hospitalisations (overall and due to heart failure), NYHA stage, treatment, adherence, weight and obesity between the groups with longitudinal random-effects models described previously. Estimation methods developed by Verbeke and Molenberghs will be used to account for missing data (GPs and patients) [25].

### Ethical considerations

The Institute’s ethics committee (Comité de Protection des Personnes Sud-Est I) approved the trial protocol on April 19th, 2010. The French drug agency, (Agence Nationale de Sécurité du Médicament et des produits de santé), gave its approval for the trial on March 5th, 2010 with the reference 2009-A01142-55. The study is conducted in compliance with the regulations on patient confidentiality (Comité consultatif sur le traitement de l’information en matière de recherche dans le domaine de la Santé (Advisory Committee on Data Processing for Matters of Research in the Field of Healthcare) and Commission Nationale de l’Information et des Libertés (National Commission for Data Protection) agreements under the reference 1223379). In compliance with the extended CONSORT statement for cluster, ETIC was registered on ClinicalTrials.gov as NCT01065142 [26].

## Discussion

ETIC is one of the few trials that involving only GPs [27]. Although multidisciplinary patient education programmes are probably more effective [28], the current healthcare organisation in France makes large-scale implementation of this type of intervention difficult, if not impossible. The idea behind ETIC is to evaluate a complex intervention including GP’s 2-day workshop and patient’s education programme in primary care in the current context of healthcare organisation in France to implement education into daily routine.

In Europe, most of the published trials regarding patient education programmes involve hospitalised or younger patients with a profile which is different from patients seen in primary care [29]. In a meta-analysis of randomised controlled trials involving patients with heart failure [30], the mean age of the patients was 61.4 years. In a recent Italian epidemiological study of primary care of patients with chronic heart failure, the mean age was 78.5 years [31]; our study population will probably have a comparable age.

In the ETIC trial, two different quality of life questionnaires are being used, one generic, the SF-36, and the other specific for chronic heart failure, the MLHFQ. The SF-36 questionnaire was chosen because the French version has been validated and it evaluates both physical and mental aspects of the quality of life, taking into consideration cultural differences [16,32]. The MLHFQ questionnaire was chosen because it is specific for patients with CHF, it is also validated in French and it accommodates psychometric properties [33]. In addition, it is recommended for initial use in clinical research [34], has been validated in primary care and is simple and fast to use [17].

The intervention in ETIC is novel because it is standardised with the topics generally used (clinical alarm signs, physical activity, diet and cardiovascular risk factors) [35], but it also involves a holistic approach, centred on the patients’ needs and feelings and taking into consideration their comorbidities and the representations of their disease [36]. The first and subsequent patient education sessions are based on, respectively, educational diagnosis and changes in habits, desires and worries about discussing specific topics, such as smoking habits [21,37].

For instance, one study on 240 patients with a mean age of 70 years and a majority of NYHA stage III (96.4%) reported baseline SF-36 mean scores of 45.1 in the intervention group and 46.8 in the control group [14]. The baseline MLHFQ mean scores were 42.5 and 42.6, respectively. In another study on 200 patients with a mean age of 63.5 years and a majority of patients with NYHA stage II-III (94%; 6% had stage IV), the baseline MLHFQ mean scores were 64.3 and 62.4 in the intervention and control groups, respectively [10]. In a study comparing home and hospital follow-up in 280 patients with a mean age of 71 years and slightly fewer patients with NYHA stage II-III (85%; 15% had stage IV), the baseline MLHFQ scores were 48.8 and 46.0, respectively [11]. In three other studies with 89%, 50% and 80% patients with NYHA stage III or IV, the mean MLHFQ scores were 49.3, 51.8, and 44.7, respectively [12,13,23]. Our patients will probably be older, with a better quality of life than the patients in all these studies because they are recruited in a primary care setting with NYHA stage I, II or III.

To our knowledge only one other cluster-randomised trial that measured the impact of a patient education programme by GPs has been published [23]. A total of 283 patients (mean = 76.3 years) were included: stage I – 1.8%; stage II – 7%; stage III – 38.9%; and stage IV – 50.5%. The mean MLHFQ score was 49.1 and 49.9 in the intervention and control groups, respectively.

The frequency of the visits in the ETIC trial, every 3 months, was similar to the standard of care; 87% of patients with heart failure meet their GP at least four times a year [38]. However, the educational aspects of the visits can be difficult for some of the GPs in the intervention group.

This study has limitations. The GP randomisation was done before the patient inclusions. The patients in the two groups might be different because the training received by the GPs in the intervention group may make them feel more competent and therefore, more inclined to include more severely ill patients. To avoid this bias, we could recruit randomised GPs after they had included their patients, using the ‘Zelen’ method [39]. Unfortunately, this has not been possible because the inclusion period lasts one year and the follow-up 19 months; regarding heart failure patient life expectancy, it would not be relevant [5].

GPs participating in this study might be interested in patient education and not be representative of French GPs. This bias is inherent in any study because GPs must volunteer to participate. Otherwise, it would be possible that the usual care group could modify their medical practice, due to the observation, which is known as Hawthorne effect [40].

The power of this study might be too low to detect differences in mortality, hospitalisations and secondary outcomes. Our interpretation will not focus only on statistical significance but on the effect size and the magnitude of improvement describing the clinical relevance [41]. Therefore, the statistical analysis plan includes multivariate analyses with adjustment for differences in the characteristics between the two groups.

In France, some studies have assessed the impact of patient education for CHF patients [7,8]. However, these interventions were delivered from multidisciplinary teams, associated in part with the hospital. This trial will provide insight in the effectiveness of a complex intervention including a GP’s 2-day workshop and patient’s education programme in the setting of a GP consultation to improve the quality of life in patients with chronic heart failure in a pragmatic approach. This complex intervention could be a CHF management tool for use during initial and further medical training.

### Trial status

The ETIC study is on-going.

## Additional files

Additional file 1:
**Clinical alarm signs and dietary leaflets.** These leaflets are given to patients. Additional file 2:
**First educational session.** This document is in case report form. Additional file 3:
**Educational sessions summary.** This document is in case report form.

## Abbreviations

BMI: Body mass index
CHF: Chronic heart failure
HF: Heart-failure
HFpEF: Heart failure with preserved ejection fraction
HFrEF: Heart failure with reduced ejection fraction
GP: General practitioner
NYHA: New York Heart Association
MLHFQ: Minnesota Living with Heart Failure Questionnaire
SF-36: MOS 36-Item Short Form Health Survey
