# Supplementary material for: Patient education in chronic heart failure in primary care (ETIC) and its impact on patient quality of life: design of a cluster randomised trial
Source: BMC Fam Pract. 2014 Dec 24;15:208. doi: 10.1186/s12875-014-0208-3 (PMC4305249; doi:10.1186/s12875-014-0208-3)
Supplement: Additional file 1: — Clinical alarm signs and dietary leaflets. These leaflets are given to patients. [file 12875_2014_208_MOESM1_ESM.pdf]

Clinical alarm signs

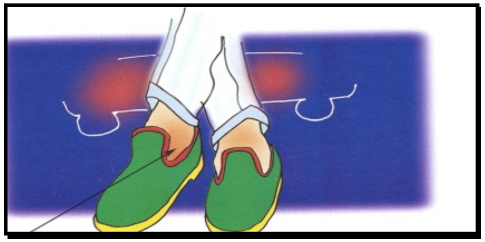

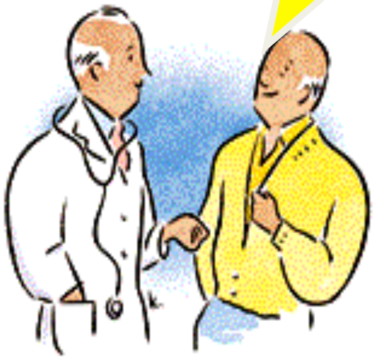

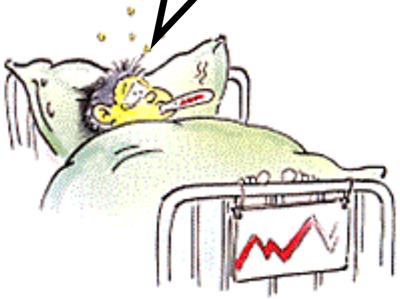

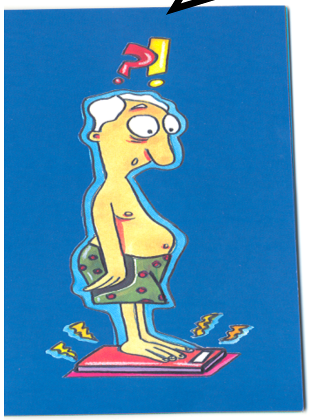

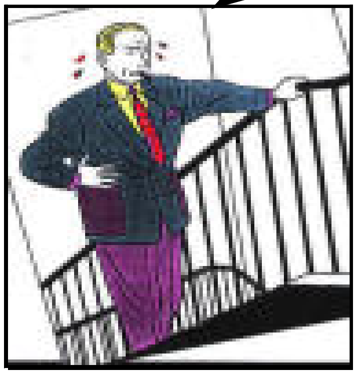

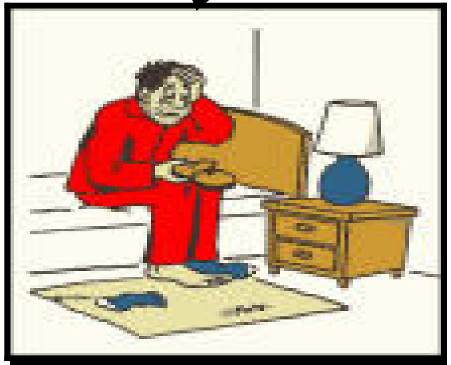

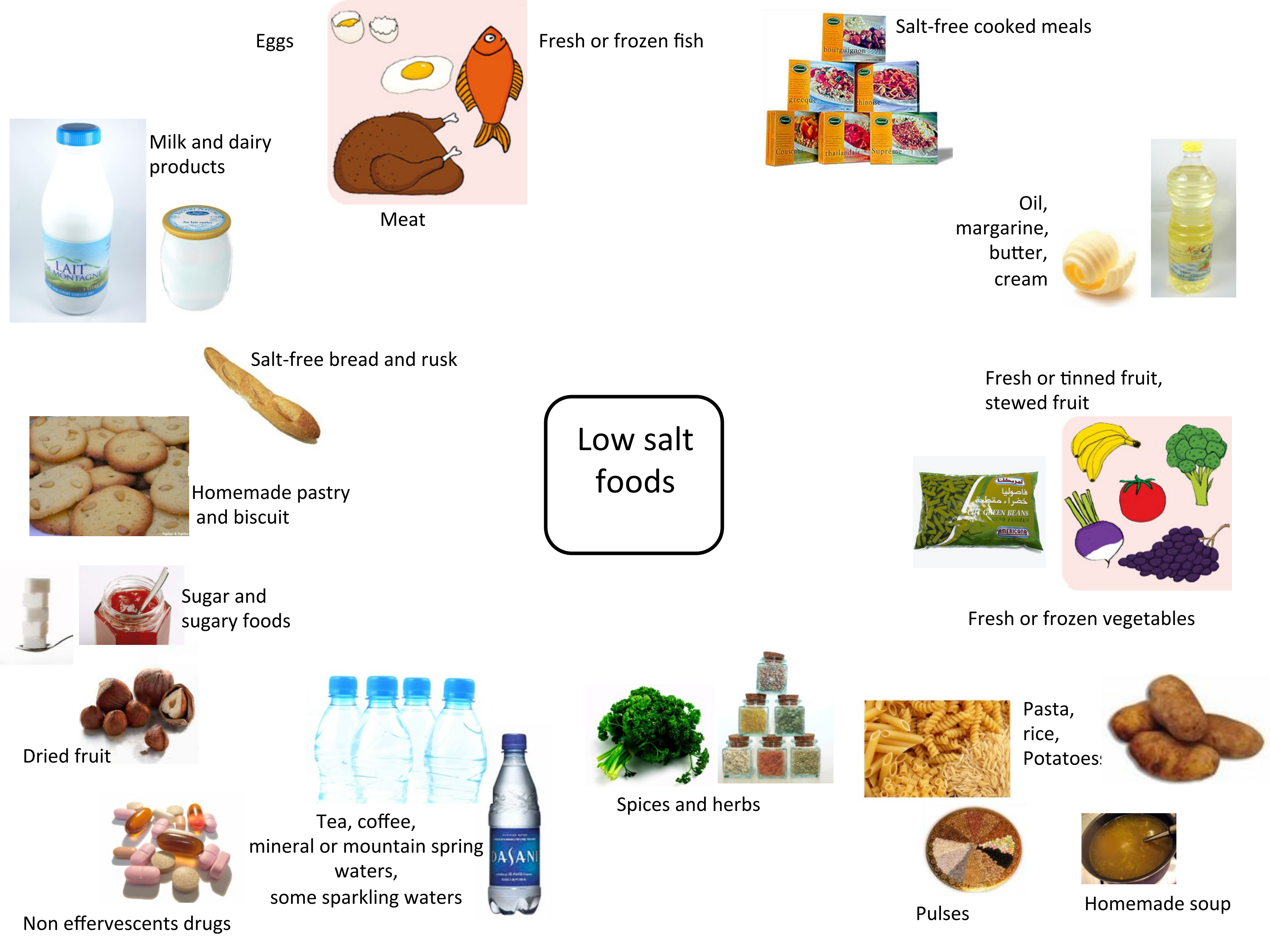
Low salt foods
Eggs
Fresh or frozen fish
Salt-free cooked meals
Milk and dairy products
LAIT DE MONTAGNE
Meat
Oil, margarine, butter, cream
Salt-free bread and rusk
Fresh or tinned fruit, stewed fruit
Homemade pastry and biscuit
Sugar and sugary foods
Fresh or frozen vegetables
Dried fruit
Spices and herbs
Pasta, rice, Potatoes
Tea, coffee, mineral or mountain spring waters, some sparkling waters
DASANI
Non effervescents drugs
Pulses
Homemade soup

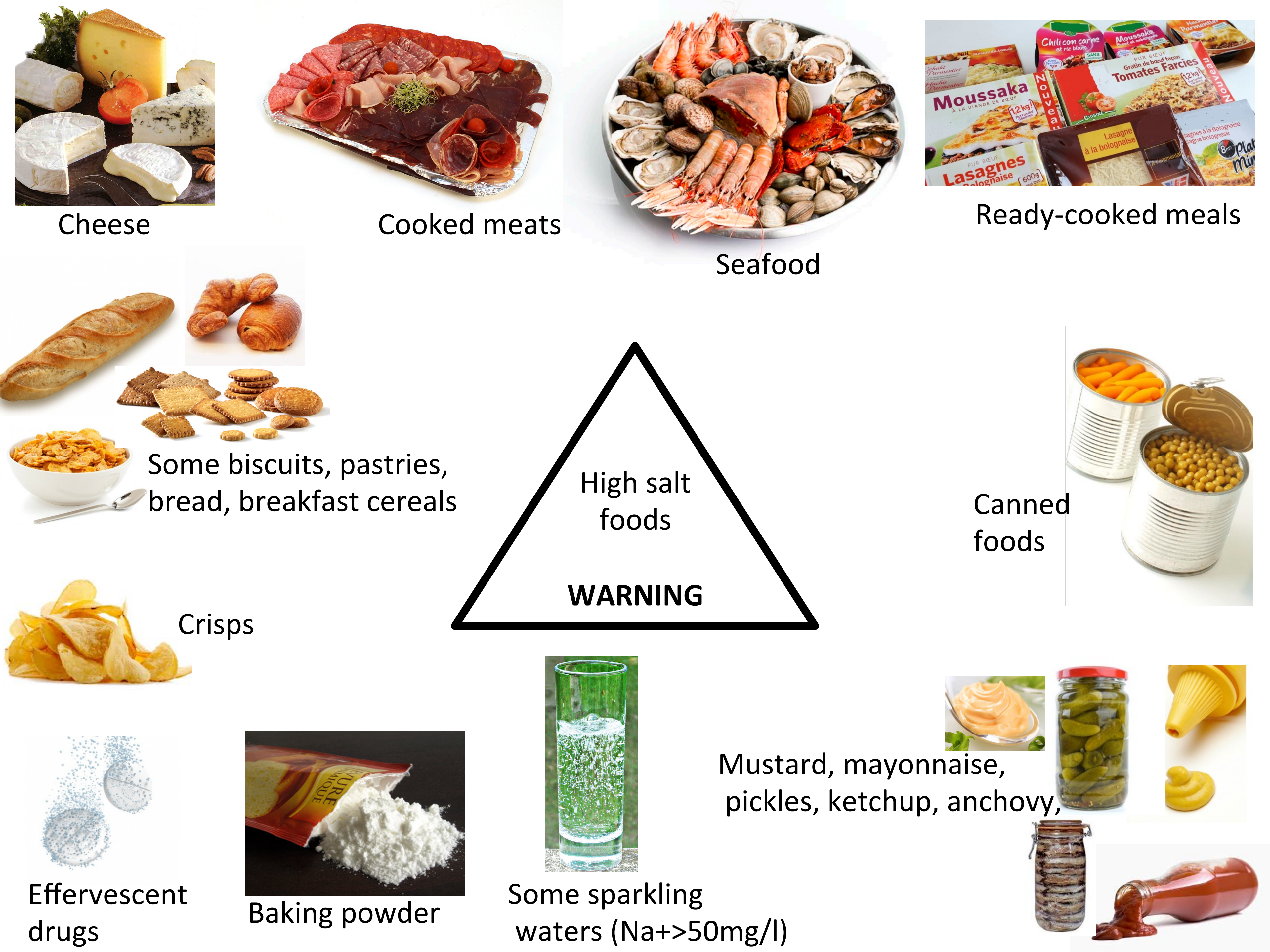
Cheese
Cooked meats
Seafood
Ready-cooked meals
Some biscuits, pastries,
bread, breakfast cereals
High salt
foods
WARNING
Canned
foods
Crisps
Mustard, mayonnaise,
pickles, ketchup, anchovy,
Effervescent
drugs
Baking powder
Some sparkling
waters (Na+>50mg/l)
