# Supplementary material for: Patient education in chronic heart failure in primary care (ETIC) and its impact on patient quality of life: design of a cluster randomised trial
Source: BMC Fam Pract. 2014 Dec 24;15:208. doi: 10.1186/s12875-014-0208-3 (PMC4305249; doi:10.1186/s12875-014-0208-3)
Supplement: Additional file 2: — First educational session. This document is in case report form. [file 12875_2014_208_MOESM2_ESM.pdf]

Main care-giver	General practitioner	Cardiologist	Nurse
Last name:	Last name:	Last name:	Last name:

First education session at one month

Year of heart failure diagnosis: …………….

Current health problems:...

- ❑ Diabetes ❑ Dyslipidaemia ❑ Hypertension
- ❑ Tobacco: ❑ Alcohol

**Life-style:**<br>Place of living:<br><br>Lives with:<br><br>Carers:<br><br>Profession (current or retired):	**Physical activity:**<br>Household:<br><br>Leisure (e.g. gardening):<br><br>Transportation (e.g. walking, car):
**Eating habits:**<br>Where meals are eaten:<br><br>Who cooks? :<br><br>Eats with:<br><br>Consumption of high salt foods:	**Hobbies, leisure activities, projects:**<br>-<br>-<br>-<br>-<br>-
**Knowledge, attitudes and motivation:**	
