# Supplementary material for: Patient education in chronic heart failure in primary care (ETIC) and its impact on patient quality of life: design of a cluster randomised trial
Source: BMC Fam Pract. 2014 Dec 24;15:208. doi: 10.1186/s12875-014-0208-3 (PMC4305249; doi:10.1186/s12875-014-0208-3)
Supplement: Additional file 3: — Educational sessions summary. This document is in case report form. [file 12875_2014_208_MOESM3_ESM.pdf]

Date :<br>………………..	Resources and levers	Difficulties
NYHA stage:		
Current treatment<br>-<br>-<br>-<br>-<br>-<br>-<br>-<br>-<br>-<br>-<br>-<br><br>weight (Kg) :<br>Height (m) :<br>BMI (Kg/m2):		

SYNTHESIS

Personal objectives

-……………………………………..

-……………………………………..

-……………………………………..

❑ Synthesis validated by the patient

During the consultation, did you discuss about :

	Yes	No
Adherence to treatment	1 ❑	2 ❑
Dietary …..	1 ❑	2 ❑
Physical activity	1 ❑	2 ❑
Tobacco...	1 ❑	2 ❑
Clinical alarm signs………………………………………..…………………………….….	1 ❑	2 ❑

Others :

………

………

………

……………………………………………………………………………
